# Neural Progenitor Cell Polarity and Cortical Development

**DOI:** 10.3389/fncel.2017.00384

**Published:** 2017-12-05

**Authors:** Yoko Arai, Elena Taverna

**Affiliations:** ^1^Centre for Interdisciplinary Research in Biology (CIRB), Collège de France, CNRS UMR 7241/INSERM U1050, PSL Research University, Paris, France; ^2^Department of Evolutionary Genetics, Max Planck Institute for Evolutionary Anthropology (MPG), Leipzig, Germany

**Keywords:** brain development, epithelial polarity, apical progenitors, basal progenitors, neural stem and progenitor cells, polarity, epithelial to mesenchymal transition (EMT), neurodevelopmental disorders

## Abstract

Neurons populating the cerebral cortex are generated during embryonic development from neural stem and progenitor cells in a process called neurogenesis. Neural stem and progenitor cells are classified into several classes based on the different location of mitosis (apical or basal) and polarity features (bipolar, monopolar and non-polar). The polarized architecture of stem cells is linked to the asymmetric localization of proteins, mRNAs and organelles, such as the centrosome and the Golgi apparatus (GA). Polarity affects stem cell function and allows stem cells to integrate environmental cues from distinct niches in the developing cerebral cortex. The crucial role of polarity in neural stem and progenitor cells is highlighted by the fact that impairment of cell polarity is linked to neurodevelopmental disorders such as Down syndrome, Fragile X syndrome, autism spectrum disorders (ASD) and schizophrenia.

## Introduction

The cerebral cortex is the center of higher cognitive functions. Neurons and glial cells populating the cerebral cortex arise sequentially during embryonic development from the division of neural stem and progenitor cells.

Polarity, that is the asymmetric spatial organization of cellular components and subcellular structures, is one of the main criteria used to classify and distinguish different types of stem and progenitor cells (Fietz and Huttner, 2011; Taverna et al., 2014). In actively dividing cells, such as neural stem cells, the polarity cues of the mother cells are used to generate different types of daughter cells: polarity is therefore instrumental in increasing cell type diversity (Fietz and Huttner, 2011). This is a crucial aspect in the central nervous system (CNS), particularly for cerebral cortex development and evolution, as enhanced cognitive functions in mammals are thought to arise from an increase in the diversity of cell types; in particular neural progenitor cell types in the developing cerebral cortex (Wilsch-Bräuninger et al., 2016; Arai and Pierani, 2014).

In this review article, we will focus on: (i) neural stem and progenitor cells and their polarity features; (ii) molecular mechanisms underlying neural stem and progenitor cell polarity; (iii) cell polarity and cell identity; and (iv) how the impairment of cellular polarity impacts cortical development.

## Neurogenesis in Mammals: Cell Types and Their Polarity Features

**Figure 1:**
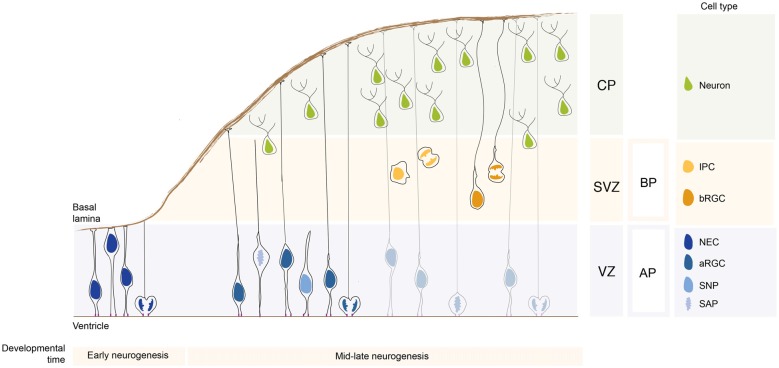
Neural stem and progenitor cell types in the developing neocortex. During early neurogenesis, neuroepithelial cells (NECs) form the ventricular zone (VZ) and are responsible for the lateral expansion of the neocortex. During mid-late neurogenesis, apical progenitors (APs) divide and give rise to basal progenitors (BPs), which form a new proliferative zone, the subventricular zone (SVZ). The APs pool is mainly composed of apical radial glial cells (aRGCs) and a lower proportion of short neural precursors (SNPs) and sub-apical progenitors (SAPs). The BPs pool is composed by intermediate progenitor cells (IPCs) and basal radial glial cells (bRGCs). APs and BPs give rise to neurons that migrate basally and settle in the forming cortical plate (CP). The relative proportion of the different neural stem and progenitor cells changes greatly in species with different encephalyzation and gyrification. APs and BPs generate in a tightly controlled temporal order the pyramidal neurons populating the 6-layered neocortex (not depicted here for simplicity).

Neural stem cells compared to neural progenitor cells differ with regards to their multipotency state: while neural stem cells are multipotent, neural progenitor cells are unipotent and fate restricted. They are classified based on several criteria, such as the location where they undergo mitosis, polarity features, and proliferation vs. differentiation potential (Taverna et al., 2014). Based on the location of their mitoses, neural stem and progenitor cells fall into two groups: apically-dividing and basally-dividing progenitor cells apical progenitors (APs) and basal progenitors (BPs), respectively (Figure 1; note that unless specified otherwise, the findings reported here refer to the developing dorsal telencephalon of rodents).

### Apical Progenitors

The term apical stem and progenitor cells (APs) refer to cells undergoing mitosis at the apical surface of the ventricular zone (VZ; Figure 1). This category is comprised of cells which have a wide range of mitotic capacity and proliferation/differentiation potential. In the subsequent sections, we will describe the different AP subtypes in order from the most multipotent down to unipotent cells.

### Neuroepithelial Cells

Before the onset of neurogenesis, the developing brain is formed mostly by neuroepithelial cells (NECs). They are highly polarized epithelial cells exhibiting apico-basal polarity (Figure 1). Their apical plasma membrane is integrated into the adherens junctional (AJ) belt and lines the lumen of the neural tube, which is filled with lipoprotein- and membrane particle-rich embryonic cerebrospinal fluid (Lehtinen et al., 2011). The AJs are cell junctions surrounding the cell, they are linked to the actin cytoskeleton and they separate the apical and the basal domain of NECs (for an historical perspective on AJs refer to Franke, 2009; and references therein; see also Farquhar and Palade, 1963; Takeichi, 1977; Stocker and Chenn, 2015). The basal plasma membrane of NECs spans the neuroepithelium and reaches the basal lamina, a rich source of extracellular molecules (Vaccarino et al., 1999a,b; Raballo et al., 2000; Götz and Huttner, 2005; Fietz et al., 2010). This highly dynamic and rich micro-environment provides a “stem cell niche” to the NECs during development (Lehtinen et al., 2011) that is crucial for the regulation of neurogenesis. NECs undergo interkinetic nuclear migration (INM), that is, they move their nuclei in the VZ in concert with the cell cycle: after completing mitosis at the ventricular surface, their nuclei undergo apical-to-basal migration during G1. After exiting from S-phase at the basal part of the VZ the nuclei undergo basal-to-apical migration, so that their successive mitosis will occur again at the ventricular surface (see Taverna and Huttner, 2010; Lee and Norden, 2013 and references therein). The NECs mitosis is confined to the ventricular surface, as the apical plasma membrane harbors the primary cilium that is nucleated by the centrosomes that also builds the mitotic spindle (see Taverna and Huttner, 2010; and references therein). NECs undergo proliferative divisions to expand the NEC pool. Ultimately, they develop into radial glial cells. Although it is not the main topics of this review article, it is important to mention that a proportion of NECs are embryonic neural stem cells from which adult neural stem cells originate (Furutachi et al., 2015). Using a strategy to follow the cell cycle progression of NECs, it has been shown that a subpopulation of NECs at early developmental stage gives rise to adult neural stem cells. This NECs subpopulation can be therefore considered as an embryonic neural stem cell (Furutachi et al., 2015). Embryonic neural stem cells slow down their cell-cycle speed allowing cells remain in a quiescence state. It still remains unclear how this embryonic neural stem cell population is determined during development and if adult neural stem cells can have also a radial glial origin.

### Apical Radial Glial Cells

**Figure 2:**
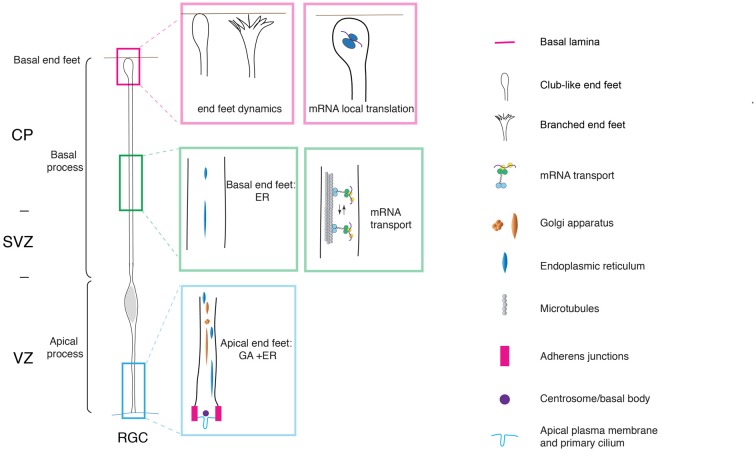
Cell polarity and subcellular dynamics in apical radial glial cells. Apical radial glial cells (aRGCs) are highly polarized along their apico-basal axis. The adherens junctions maintain aRGC architecture and tissue integrity at the apical end feet. The basal process contacts the basal lamina with the basal end foot, where several mRNAs are transported and locally translated. The morphology of the basal end feet changes dynamically during neurogenesis. An organelle such as Golgi apparatus (GA) is distributed in the apical process exclusively. On the other hand, the endoplasmic reticulum (ER) is distributed globally in basal and apical processes. aRGC, apical radial glial cell; CP, cortical plate; SVZ, sub-ventricular zone and VZ, ventricular zone (the drawing is adapted from Taverna et al., 2016, licensed under a Creative Commons Attribution 4.0 International License).

With the onset of neurogenesis, NECs differentiate into apical radial glial cells (aRGCs; Malatesta et al., 2003; Götz and Huttner, 2005). aRGCs are even more elongated than NECs. Their basolateral plasma membrane is divided in two sub-compartments: the apical and the basal process (Figures 1, 2). The apical process is the portion of the basolateral plasma membrane residing in the VZ and it accommodates the nucleus during the different phases of INM. The basal process is the portion of the cell that traverses the sub-ventricular zone (SVZ) and the forming neuronal layers and reaches the basal lamina. Of note, as neurogenesis proceeds and neuronal layers are formed, the width of the cortical wall increases radially: therefore, the basal process elongates. The basal process functions as a guide for radial neuronal migration, allowing newborn excitatory cortical neurons to translocate from the place of birth to their final destination. In addition to providing a migratory scaffold for neurons in their journey to the cortical plate (CP), the basal process is a subcellular compartment involved in signaling and fate specification (Stenzel et al., 2014). Furthermore, live imaging has revealed that the basal process is a very dynamic entity, with the basal end changing from highly branched to club-like during cortical development (Yokota et al., 2010; Figure 2).

aRGCs have been extensively studied in the last decades and it is now clear that they contribute to neurogenesis mainly via the generation of a second type of neural progenitor cells: the BPs (Pontious et al., 2008; Kowalczyk et al., 2009). Interestingly, one of the main difference between apical and BPs is the absence of apical polarity cues in the latter.

### Short Neural Precursors

Short neural precursors (SNPs, also known as apical intermediate progenitors) were first described in the mouse developing neocortex, where they exhibit several features in common with aRGCs, such as the bipolar morphology and the integration into the AJ belt (Gal et al., 2006; Tyler and Haydar, 2013). Unlike aRGCs, SNPs feature a basal process that does not traverse the neuronal layer, but it is confined to the VZ (Figure 1). A potentially similar cell type was reported to be present in the mouse ventral telencephalon, where aRGCs give rise to interneurons (Tan et al., 2016). As development proceed, the aRGCs in the ventral telencephalon grow radial glial fibers that no longer reach the basal lamina, but rather contact periventricular vessels (Tan et al., 2016). The vessel-anchored aRGCs undergo INM, divide apically (as SNPs do) and maintain the radial fiber throughout mitosis (unlike SNPs). Furthermore, a recent paper (Nowakowski et al., 2016) shows that in the human developing neocortex, during the mid-neurogenesis stage, aRGCs transform into “truncated” aRGCs, with a basal process that no longer reaches the basal lamina, but terminates in the depth of the cortical wall. Several interesting questions remain: are SNPs, vessel-anchored aRGCs in the mouse ventral telencephalon and truncated aRGCs in human related? Is the lack of basal attachment affecting the radial migration of the daughter cell after division? Is the daughter cell migrating for shorter distances?

### Subapical Progenitors

Subapical progenitors (SAPs) were identified in the mouse ventral telencephalon and in the dorsal telencephalon of gyrencephalic species (Pilz et al., 2013). SAPs are anchored to the ventricle with an apical process. However, they undergo mitosis at a subapical location (Pilz et al., 2013; Figure 1). Most likely, in SAPs the centrosome is not docked at the apical plasma membrane and is therefore free to move basally to nucleate the mitotic spindle, as opposed to what happens in NECs where the centrosome is restricted to the apical side (see above).

### Basal Progenitors

The term BPs indicates cells which undergo mitosis in the SVZ, the secondary germinal zone located basally compared to the VZ (Figure 1). BPs are generated by divisions of aRGCs and move basally via a process known as delamination. BPs are further divided into two classes: the intermediate progenitors (IPCs) and the basal radial glial cells (bRGCs).

### Intermediate Progenitor Cells

IPCs represent the main class of BPs in rodents, as originally described independently by three different groups (Haubensak et al., 2004; Noctor et al., 2004; Miyata et al., 2004). IPCs are non-polar cells, as they lack both apical and basal polarity cues (Figure 1). The process of delamination of an IPC from the apical junctional belt very much resembles an epithelial-to-mesenchimal transition (EMT; Wilsch-Bräuninger et al., 2016), in which an epithelial cell gives rise to an unpolarized, highly motile cell (reviewed in Acloque et al., 2009; Itoh et al., 2013b). EMT is a process by which a polarized epithelial cell transforms into an unpolarized and highly motile mesenchymal cell. EMT occurs extensively during embryogenesis and it is crucial for gastrulation and neural crest formation. In pathological conditions, EMT is associated with in the initiation of metastasis and with cancer progression (Acloque et al., 2009; Itoh et al., 2013b; Wilsch-Bräuninger et al., 2016).

### Basal Radial Glial Cells

Basal radial glial cells were first described in the developing neocortex of gyrencephalic species, namely in humans and ferrets, and were subsequently found, albeit at a much lower abundance, also in the lissencephalic developing rodent brain (Fietz et al., 2010; Hansen et al., 2010; Reillo et al., 2011; Wang et al., 2011; Reillo and Borrell, 2012; Sauerland et al., 2016). From an evolutionary perspective, the pool of bRGCs greatly expanded in humans and other gyrencephalic species, leading to the generation of two separate basal germinal zones: the inner and outer sub VZ (ISVZ and OSVZ, respectively; Smart et al., 2002). Recently, bRGCs have attracted great interest, as their abundance seems to correlate with the extent of brain expansion across species and with gyrification (Reillo et al., 2011; Kelava and Huttner, 2013). Several lines of evidence, including wide occurrence of bRGs in the marsupials and wallaby, suggest that bRGs might have been present in the ancestor of all mammals (Kelava et al., 2012; Sauerland et al., 2016). From a cell biological point of view, bRGCs are monopolar cells lacking an apical attachment (Figure 1). Interestingly, bRGCs still maintain an attachment to the basal lamina via a basal process (Hansen et al., 2010; Florio et al., 2015; Nowakowski et al., 2016). Functional manipulation has shown that the basal process is crucial for the maintenance of the proliferative capacity of bRGCs (Fietz et al., 2010). bRGCs appear to be a rather heterogeneous cell population, as shown by high resolution live imaging of the developing macaque neocortex (Betizeau et al., 2013). The heterogeneity was found to be both morphological and transcriptional. In particular, macaque bRGCs differ in term of presence vs. absence of apical-directed and basal-directed processes (Betizeau et al., 2013).

In summary, APs and BPs show striking differences in cell polarity. Evidences are accumulating that polarity influences the behavior of neural stem and progenitor cells during brain development.

## Molecular Mechanisms of Neural Progenitor Cell Polarity

Key players of the maintenance of aRGCs polarity were found to be localized at the apical and basal end foot. The apical end foot of aRGCs is composed of the apical plasma membrane and the AJ belt (Figure 2). The apical plasma membrane represents a minor fraction of the total plasma membrane (1%–2%) and features the primary cilium that protrudes in the lumen of the ventricle and receives signals generated therein. The apical plasma membrane is delimited by the AJ, a subcellular structure that plays a crucial role in maintaining aRGCs architecture and function at the apical pole. At the cellular level, the AJ separates the apical and basolateral plasma membrane, while at the tissue level it maintains the integration of aRGCs in the neuroepithelium, securing tissue integrity. Consistent with this notion, perturbation of AJ components and polarity proteins localized at the apical end foot results in severe changes in APs morphology and function (Chenn and Walsh, 2002, 2003; Cappello et al., 2006, 2012; Katayama et al., 2011; Durak et al., 2016).

Among the polarity proteins regulating aRGCs function, Cdc42 represents a very interesting case, as it regulates the structure and function of aRGCs both at the apical and the basal pole. Indeed, the loss of function of Cdc42 results in the gradual loss of AJs and retraction of the apical processes, ultimately leading to an increase in the generation of IPCs and in turn premature neuronal differentiation (Cappello et al., 2006). Interestingly, the manipulation of Arp2/3 complex, a downstream effector of Cdc42, shows a phenotype similar to the Cdc42 mutant mouse, with altered aRGCs polarity, defective adhesion and an increased number of IPCs. Arp2/3 is an actin nucleator producing branched actin networks, suggesting a possible involvement of the actin cytoskeleton in maintaining aRGCs polarity. The actin cytoskeleton was found to be involved in the G1 apical-to-basal phase of INM in aRGCs and the pharmacological inhibition of actin contractility led to an increase in basal mitoses in the developing mouse neocortex (Schenk et al., 2009), suggesting a role for actin in maintaining a progenitor pool. The actin cytoskeleton is also crucial for vesicle trafficking, exocytosis and endocytosis. The formation of AJs requires the transport of cadherins and apical polarity proteins from trans-Golgi networks to the plasma membrane (Sheen et al., 2004). Furthermore, endocytosis may allow for the recycling of cadherins and the dynamic remodeling of the AJs in response to a change in aRGCs activity and function.

Cdc42 was found to be localized at the aRGCs basal pole, where it regulates the dynamics of the basal end feet (Yokota et al., 2010). As revealed by live imaging experiments, the basal end foot is a very dynamic structure, with small protrusions emanating from the basal process shaft, possibly engaging in cell-to-cell communication between aRGCs. The expression of the Cdc42 dominant negative results in morphological changes in the basal end feet, with a concomitant reduction in inter-radial glia interaction (Yokota et al., 2010). Of note, and consistent with the fact that Arp2/3 is a downstream effector of Cdc42, Arp2/3 was found to have an effect on the dynamics of the basal process. Upon conditional ablation of the Arp2/3 complex in aRGCs, the dynamics of basal process extension is altered and this ultimately results in an overall change in organization, orientation and length of the basal process (Wang et al., 2016).

## Cell Polarity and Subcellular Dynamics

The extreme elongation of aRGCs and the division of the basolateral plasma membrane in apical and basal process pose very interesting questions. Is the subcellular organization different between the apical and the basal process? How do intracellular organelles, in particular the biosynthetic pathway, help build the apical and basal process? Using a panel of morphological approaches, it was recently shown that in aRGCs the Golgi apparatus (GA) is strongly polarized along the cell’s apico-basal axis, and is confined to the apical process (Taverna et al., 2016; Figure 2). In contrast, the endoplasmic reticulum (ER) was found to be present in both the apical and the basal process. The confinement of the GA to the apical process of aRGCs impacts on the composition of the apical and basal process plasma membrane: the basal process plasma membrane was found to contain almost exclusively ER-derived glycans, whereas the apical process contains both ER- and Golgi-derived glycans. These observations prompted the authors to propose that the biosynthesis of the apical and basal process could rely on two different mechanisms: the delivery to the apical process plasma membrane is thought to largely occur via the canonical biosynthetic route (ER → GA–plasma membrane), whereas the delivery to the basal process plasma membrane has been proposed to occur via an unconventional route that bypasses the GA (ER → plasma membrane; Taverna et al., 2016; Figure 2).

What underlies the confinement of Golgi-derived glycans to the apical process? In neurons, the axon initial segment confines proteins and lipids to the axonal plasma membrane, thus contributing to maintain the identity of the axo-dendritic compartments (Rasband, 2010). One can speculate that a similar macromolecular complex could be involved in creating a boundary between the apical and the basal process, creating a diffusion barrier for membrane-bound and cytoplasmic molecules.

The enrichment of ER-derived glycans in the basal process plasma membrane may provide a specific environment for neuronal migration. Several questions remain to be answered. Do the particular glycans present in the membrane of the aRGCs have an influence on the behavior of the IPCs? Are ER-derived glycans influencing the migratory kinetics of neurons in their long journey along the basal process? Do early-born neurons, generated from less elongated aRGCs through IPCs, containing less ER-derived glycans than late-born neurons, generated from more elongated aRGCs? What is the glycan composition of the bRGC basal process?

Another important question is if any other organelle is asymmetrically distributed between apical and basal process. Interestingly, recent data has shown that mitochondria undergo extensive morphological changes in the developing neural tube of chick and mouse embryos. This study showed that mitochondria are thick and short in interphase APs, while they are thin and strongly connected in networks in neuronal cells (Mils et al., 2015).

Not only intracellular organelles but also *mRNAs* are distributed in a highly-polarized fashion along the apico-basal axis in aRGCs, a finding that opens an exciting avenue in the field of neural stem cell biology. In particular, it was first demonstrated that the *CyclinD2 mRNA* is highly enriched at the basal end foot (Tsunekawa et al., 2012), where it is locally translated into protein (Pilaz et al., 2016; Figure 2). This finding reveals that local translation can take place far away from the VZ, the germinal zone where the cell body and nucleus resides, even for proteins exerting their action in the nucleus, as is the case for CyclinD2. Possibly, the local translation of *CyclinD2* serves as a mechanism to strictly confine in space and time the function of CyclinD2 itself. Recently, FMRP were identified as the molecular motor responsible for *mRNA* transport to and localization at the basal end foot (Pilaz et al., 2016; Pilaz and Silver, 2017; Figure 2). The authors conducted an elegant and thorough characterization of the *mRNAs* localized at the basal end foot and showed that transcripts are locally translated (Pilaz et al., 2016). The local translation is somehow reminiscent of the local translation of *mRNA* in dendrites and axons (Bramham and Wells, 2007; Lin and Holt, 2008). In the case of neurons, transcripts can be translated on demand and in an activity-dependent manner. To push the parallel further, it would be extremely interesting to understand to which extent the transport and local translation of *mRNA* in the aRGCs basal end foot is regulated in a spatiotemporal manner by cell-to-cell interaction, either between neighbors aRGCs, or between aRGCs and the surrounding basal niche formed by meninges, basal lamina and Cajal-Retzius cells.

## Cell Biological Mechanisms of APs to BPs Transition

Research in the last decade has focused on the fine cell biological mechanisms underlying APs-to-BPs fate transition and delamination (Acloque et al., 2009; Itoh et al., 2013b; Wilsch-Bräuninger et al., 2016), a process that very much resembles an epithelial-to-mesenchymal transition. Consistent with that parallel, the AJ components cadherins and catenins were found to have a role in the delamination of post-mitotic cell from aRGC and in the generation of bRGCs (Kadowaki et al., 2007; Stocker and Chenn, 2009, 2015; Itoh et al., 2013a; Martínez-Martínez et al., 2016). Conditional or focal reduction of N-Cadherin and αE-catenin, respectively, resulted in severe disruption in NECs structure and in turn affect cortical lamination (Kadowaki et al., 2007; Stocker and Chenn, 2009, 2015). Furthermore, a functional link between AJ complex and Wnt/β-catenin pro-proliferative signaling was observed in cortical progenitor cells (Hirabayashi et al., 2004; Stocker and Chenn, 2009).

One of the first detectable differences during fate transition and BP delamination is the change in the location of ciliogenesis. Cilia in APs are localized apically and they protrude in the ventricle from the apical plasma membrane, where they are tethered via the basal body (Figure 2). Elegant electron microscopy studies showed that in nascent BPs the cilium/basal body is located abventricularly, above the AJ belt (Wilsch-Bräuninger et al., 2012). The change in location of the cilium could favor cellular delamination either by favoring the “extrusion” of the apical plasma membrane from the AJ belt, or by increasing the endocytosis of the apical membrane components (though the two explanations are not mutually exclusive; Wilsch-Bräuninger et al., 2016). From a functional point of view, the relocation of the cilium could remove nascent BPs from the exposure to certain signals originating in the ventricle in favor of a signal originating in the VZ proper. An obvious question is if any other organelle undergoes reorganization upon fate transition. A good candidate in that respect is the GA, owing to the tight physical and functional link between the GA and the centrosome. Indeed, the GA in the aRGC’s apical process was found to be neither perinuclear nor pericentrosomal. Interestingly, the GA was shown to become pericentrosomal in BPs upon delamination (Taverna et al., 2016). This data suggests that upon fate transition, the lack of polarity cues induces a reorganization at the centrosome-Golgi interface.

Another organelle involved in AP-to-BP fate transition is the ER. Recent findings show a role of the ER stress and unfolded protein response (UPR) in fate transition and neurogenesis (Laguesse et al., 2015). The authors focus on Elp3, a Elongator complex protein expressed in APs, where it maintains translational fidelity through the regulation of tRNA modification. Disruption of Elp3 decreases the speed of translation, promoting ER stress response and UPR upregulation. The knockout of Elp3 shows a progressive downregulation of UPR in APs and an amplification of IPCs leading to microcephaly (Laguesse et al., 2015). In a recent report, the authors also showed a role of Elp3 in the regulation of acetylation and membrane distribution of connexin-43 (Cx-43, Gja1; Laguesse et al., 2015). Cx-43 is a gap junction component expressed in APs where it plays a crucial role in cell-to-cell communication, INM and radial migration of neurons (Pearson et al., 2004; Sutor and Hagerty, 2005; Elias et al., 2007; Liu et al., 2010).

## Cell Polarity and Cell Fate Specification: Relevance of Polarity for Neural Stem Cell Function

Cell polarity has important implications for neural stem cell fate for two main reasons: (i) the polarized organization allows progenitors to differentially respond to signals from the ventricle and/or from the basal pole; and (ii) the apical-basal polarity of aRGCs is the structural basis for their symmetric vs. asymmetric division, as defined by an equal vs. unequal distribution of cellular components to the daughter cells. Polarity is therefore instrumental in generating neural stem cell diversity.

### Polarity and Differential Responses to Apical and Basal Niches

The organization of aRGCs along their apico-basal axis somehow mirrors the histological organization of the cortical wall. In that context, the basal and apical extensions of aRGCs could allow the aRGCs to sense, integrate and respond to different signals generated in different niches, either at the apical or at the basal pole. The apical plasma membrane delimits the ventricle, which is filled with cerebrospinal fluid, and contains different signaling molecules including morphogens (Lehtinen et al., 2011, 2013). At the basal pole, the basal end feet are physically in contact with meninges, extracellular matrix and Cajal-Retzius cells which are sources for morphogens promoting proliferation and/or differentiation (Siegenthaler et al., 2009; Griveau et al., 2010). The basal end foot is therefore in a privileged position to sense and respond to basal extracellular signals. These signals could regulate local biological processes such as *mRNA* translation (Tsunekawa et al., 2012; Pilaz et al., 2016). It is interesting to note that due to their cellular organization, different neural stem cell subtypes have different level of access to signaling niches localized along the apico-basal axis of the cell and of the tissue. Since during development and evolution there is a progressive shift from apical to basal mitosis, it is tempting to speculate that this shift also represents a shift in signaling, with stem cells being regulated by basal and apical polarity cues during early development, and then being regulated mainly by basal polarity cues (Stenzel et al., 2014). This could also represent a way to restrict the expansion of the ventricular surface and favor the expansion of the basal part of the developing neocortex.

### Polarity and Cell Division

The cell polarity of APs is the structural basis for symmetric vs. asymmetric division as it allows the equal vs. unequal distribution of cellular components to the daughter cells (Huttner and Kosodo, 2005). Several findings support the idea that the apical pole and subcellular structure therein are asymmetrically partitioned and influence cell fate. The apical plasma membrane constitutes a minor proportion of the total plasma membrane and can be either bisected or bypassed by the cleavage plane, resulting in only one daughter cell inheriting a portion of the apical plasma membrane (Kosodo et al., 2004). The cell inheriting the apical plasma membrane was reported to maintain proliferative potential (Kosodo et al., 2004). Not all the apical plasma membrane is partitioned based on the cleavage plane orientation: it was shown that the ciliary membrane, a specialized domain of the apical plasma membrane, is endocytosed at the onset of mitosis (Paridaen et al., 2013). The ciliary membrane preferentially associates with the mother centriole, is conserved throughout mitosis in association with one spindle pole and is asymmetrically inherited by one of the two daughter cells (Paridaen et al., 2013). The cell inheriting the ciliary membrane re-establishes the cilium faster and tend to maintain stem cell-like characteristics. These findings strongly suggest a role for the apical pole and subcellular structures therein in maintaining and influencing the choice between proliferation and differentiation.

It has also been shown that the basal process can be asymmetrically inherited by one of the two daughter cells, with the cell inheriting the basal process tending to maintain proliferative capacities (Konno et al., 2008; Shitamukai et al., 2011). How is the inheritance of the basal process linked to the choice between proliferation and differentiation? The inherited basal process could maintain aRGCs in a proliferative state thanks to the inheritance of the basal process-localized *mRNAs* and/or receptors for growth factors. Furthermore, data showed that both aRGCs and bRGCs are able to re-grow their basal process (Hansen et al., 2010; Shitamukai and Matsuzaki, 2012; Betizeau et al., 2013; Subramanian et al., 2017). What are the differences between an inherited vs. a regrown basal process? Is the re-growth of the basal process a mechanism to bypass the limitation imposed by the asymmetric inheritance of the basal process, so that both daughter cells are equally able to respond to extracellular signals? Is the re-grown basal process featuring different receptors compared to the inherited basal process? One might speculate that the newly delivered receptors in a re-grown basal process features different post-translational modifications and/or has a different desensitization status, allowing the two daughter cells to respond differentially to the same extracellular stimuli. Further research will be required to obtain a coherent picture on the interplay between polarity, asymmetric division and cell fate specification. It would also be extremely interesting to extend the pioneering work performed on aRGCs to other polarized progenitor cells, such as bRGCs, in order to understand to which extent similar cell biological principles are used in different cell types and how they act in generating neural stem cell diversity.

## Impaired Cell Polarity as a Cause of Neurodevelopmental Disorders

Neurodevelopmental defects comprise a substantial proportion of neuropsychiatric diseases and the general consensus is that they originate from early events in brain development (Feng et al., 2000; Bond et al., 2002; Chenn and Walsh, 2002; Tsai et al., 2005; Shu et al., 2006; Xie et al., 2007; Singh et al., 2010; Birnbaum et al., 2014). We here focus on neurodevelopmental disorders that are reported to be associated with polarity defects in neural stem and progenitor cells (Chenn and Walsh, 2003; Hirabayashi et al., 2004; Sheen et al., 2004; Mao et al., 2009; Katayama et al., 2011; Durak et al., 2015, 2016).

### Down Syndrome

Down syndrome is the most common genetic cause of mental retardation. Patients diagnosed with Down syndrome showed an overall reduction of cerebrum gray matter volume (Weitzdoerfer et al., 2002) as well as a disorganized cortical lamination (Pinter et al., 2001). The reduction of Arp2/3 complex was reported in fetal Down syndrome brain and the conditional ablation of Arp2/3 complex in mice showed a reduction in neuronal number and highly disorganized cortical lamination (Wang et al., 2016). It would be important to understand to which extent the effects of Arp2/3 on the overall brain functions are due to the early effects of Arp2/3 on neural stem and progenitor cells, in particular on aRGCs (Tyler and Haydar, 2013).

### Fragile X Syndrome and Autism Spectrum Disorders

Fragile-X syndrome (FXS) is the most common form of inherited intellectual disability and it is caused by mutations in *Fragile X Mental Retardation 1* (*FMR1*) gene. Loss-of-functions of FMR protein (FMRP) showed: (i) a switch from AP to BP fate, indicating the depletion of aRGCs pool (Saffary and Xie, 2011); (ii) defects in neuronal positioning due to the misregulation of N-cadherin levels (La Fata et al., 2014); and (iii) early postnatal circuitry impairments possibly linked to abnormalities in the projection fibers (La Fata et al., 2014). Of interest, *FMR1* is also the most common single genetic cause of autism spectrum disorders (ASD; Hagerman et al., 2011; Bagni et al., 2012). In line with that, clinical crosstalk has been reported between FXS and ASD (Hagerman et al., 2011; Bagni et al., 2012). Considering the involvement of FMRP in *mRNA* transport in aRGCs (Pilaz et al., 2016; Pilaz and Silver, 2017), it would be interesting to understand if and how impaired *mRNA* transport in aRGCs contribute to the etiology of FXS and ASD (for an extensive discussion on the link between FXS, ASD, neural progenitors and cortical neurogenesis refer to Callan and Zarnescu, 2011; Packer, 2016; Marchetto et al., 2017 and references therein).

### Ciliopathies

Ciliopathies are genetic disorders of ciliary structure or function. Joubert syndrome (JS) and related disorders are ciliopathies clinically characterized by ataxia, psychomotor delay and cognitive impairment (Cantagrel et al., 2008). The classical form of JS is caused by mutations in *Arl13b*, a cilia-specific small GTPase. *Arl13b* mutations lead to an inverted apico-basal polarity of aRGCs and impair the ability of primary cilia to convey extracellular signals such as insulin-like growth factor (Igf; Higginbotham et al., 2013). Igf is highly enriched in the CSF. It would be interesting to understand if the relocation of the primary cilium from the apical to the basolateral plasma membrane upon APs to BPs fate transition (Wilsch-Bräuninger et al., 2012) changes the degree of exposure to extracellular signals generated from the CSF.

### Schizophrenia

Schizophrenia shares several common characteristics with ASD in term of behavioral, social and cognitive disturbances and also in term of genes implicated in the disease etiology (Carroll and Owen, 2009). *Disrupted In Schizophrenia* (*DISC1*) is a common susceptibility gene for those disorders and it is also associated with bipolar and mood disorders (Khanzada et al., 2017). Mutations in DISC1 gene lead to schizophrenic or depressive behavior (Clapcote et al., 2007; Mao et al., 2009; Dachtler et al., 2016). DISC1 is highly expressed in aRGCs during development and a knock-down of DISC1 showed a decreased proliferation of aRGCs and premature neurogenesis (Mao et al., 2009; De Rienzo et al., 2011; Ishizuka et al., 2011; Ye et al., 2017). The neuronal phenotypes observed are reminiscent of Cdc42 loss-of-functions (Yokota et al., 2010; Ishizuka et al., 2011). Consistent with that, DISC1 regulates aRGC proliferation through GSK3β, a downstream effector of Cdc42 (Clapcote et al., 2007; Ishizuka et al., 2011; Dachtler et al., 2016).

Taken together, these data suggest that alteration of aRGCs polarity can trigger neurodevelopmental and psychiatric disorders.

## Concluding Remarks

In this review article, we discussed the polarity features of neural stem and progenitor cells in the developing cerebral cortex and their functional implications. Research in the last decades clearly showed that polarity affects neural stem and progenitor cells, including their architecture and shape, INM, proliferation vs. differentiation potential and asymmetric cell division. Nowadays concepts derived from work in mice are finally applied in an evolutionary perspective: one notable example is provided by aRGCs in humans, where their extreme elongation matches the massive growth of the cerebral cortex. In the future, it is likely that the functions of the basal process will receive increasing attention, both in aRGCs and bRGCs. Here are few questions that in our opinion deserve attention: which are the differences between a aRGC and a bRGC basal process? How is the growth of the basal process in a single RGC coordinated with the global tissue growth? Which is the role of intracellular traffic in the basal process elongation? How are the biological functions of the basal process (e.g., mRNA translation) affected by extracellular stimuli, and how are they coordinated with the rest of the cell? We are now witnessing a very exciting time, when thanks to several technological breakthroughs we can reasonably expect that several of these questions will be answered, leading to a better understanding of cerebral cortex development and evolution.

## Author Contributions

ET and YA wrote the manuscript.

## Conflict of Interest Statement

The authors declare that the research was conducted in the absence of any commercial or financial relationships that could be construed as a potential conflict of interest.
